# Dimensions, stability, and deformability of DOPC-cholesterol giant unilamellar vesicles formed by droplet transfer

**DOI:** 10.12688/openreseurope.19149.3

**Published:** 2025-10-07

**Authors:** Elisa Roberti, Elisa Linda Petrocelli, Dario Cecchi, Stefano Palagi

**Affiliations:** 1 The BioRobotics Institute, Sant'Anna School of Advanced Studies, Pisa, Tuscany, 56127, Italy; 2 Università degli Studi di Roma La Sapienza, Rome, Lazio, 00185, Italy

**Keywords:** GUVs, droplet transfer, cholesterol, artificial cells, microrobots, deformability

## Abstract

**Background:**

Understanding cell membrane-like lipid bilayers is crucial for studying fundamental biological mechanisms. Giant Unilamellar Vesicles (GUVs) are key tools for this investigation and have applications in both synthetic biology and, more recently, in microrobotics. The effects of cholesterol, a key component of cellular membranes, on synthetic phospholipid membrane models like GUVs are however not fully understood, as they may vary with lipid composition and production method.

**Methods:**

We examined the size distribution, temporal stability and deformability of GUVs prepared with the droplet transfer method using different Dioleoylphosphatidylcholine (DOPC) to cholesterol ratios in the oil phase (namely 100:0, 85:15, 71:29, 60:40). Phase-contrast microscopy assessed size and stability, while deformability was tested by loading the GUVs with an aqueous ferrofluid and applying a uniform magnetic field to induce their elongation. Image analysis was conducted using Fiji and a custom Julia script.

**Results:**

The median diameters increased with the content of cholesterol, together with the dimensional distribution. In terms of stability, cholesterol generally reduced GUV median diameter over time, while it varyingly influenced the number of vesicles. As for deformability, beyond the expected elongation dependent on the intensity of the applied magnetic field, there were no statistically significant differences in GUV deformability in the presence or absence of cholesterol.

**Conclusions:**

Our findings suggest that cholesterol can lead to increased average diameter of GUVs made with DOPC through droplet transfer, while varyingly affecting their time-stability and not affecting their deformability. This study shows how small adjustments on a straightforward protocol like the droplet transfer method, provide a simple and effective way of tailoring GUV properties. Edits in the oil phase enable precise tuning of GUV membranes providing a tool for both fundamental studies and applications such as artificial cells or microrobots.

## Plain language summary

Cell membranes play a key role in the overall functioning of cells and their environmental interactions. For a better understanding of their mechanisms, a common tool is represented by Giant Unilamellar Vesicles (GUVs). These micrometric vesicles are produced through the supramolecular assembly of phospholipids, the main molecule of cell membranes, and used to explore basic biological processes, build synthetic cells, and even design microrobots. Here we study the effects that cholesterol, a key component of natural cell membranes, has on GUVs’ membrane properties. While these can vary with the production method and the types of lipids, in this study we focus on one of the most widely adopted methods (known as droplet transfer) and one of the most common phospholipids (dioleoylphosphatidylcholine or DOPC). Our results suggest that the effect of cholesterol on the size and stability of GUVs depends on its concentration, while their flexibility seems not to be affected. By exploring these properties, we aim to deepen our understanding of cell membranes and improve the design of synthetic systems for future applications.

## Introduction

Understanding cell lipid bilayers is crucial for deciphering fundamental biological mechanisms, considering their central role in various cellular processes. Insights into lipid bilayer behaviour enhance our understanding of general cellular mechanisms and the functional diversities among different cell types. To this end, synthetic lipid bilayers have long served as a fundamental tool to model biological membranes ( Dimova, 2019; Kahya & Schwille, 2006; Laurence *et al.*, 2022; Menger & Keiper, 1998; Toparlak & Mansy, 2019; Wubshet & Liu, 2023) and these supramolecular assemblies are also of great interest from an application perspective. Due to their selective permeability and ease of fabrication, lipid bilayers have been utilised in synthetic biology for the past two decades ( Robinson *et al.*, 2021; Van de Cauter *et al.*, 2023) and, more recently, in the field of microrobotics ( Sato *et al.*, 2017; Vutukuri *et al.*, 2020). Giant Unilamellar Vesicles (GUVs), liposomes with diameters ranging from 1 to 100 micrometres, have a single phospholipid bilayer that is commonly used as a lipid membrane model for characterization studies and technological applications.

In cells, the composition of the plasma membrane varies according to the cell function ( Casares *et al.*, 2019), but also in response to pathological ( Woodman & Kim, n.d.), nutritional ( Agostoni *et al.*, 2013), and pharmacological treatment conditions ( Escribá *et al.*, 2015). Characterizing the effects of variations in the composition of lipid bilayers can yield valuable insights into cell membrane behaviour, while also facilitating applications such as vesicle-based artificial cells and microrobots. Whether the goal is to understand cell membrane behaviour or to develop vesicle-based microrobots and artificial cells, it is crucial to examine how the membrane composition influences the dimensions, stability, and deformability of GUVs. One important property influencing the mechanical behaviour of cells and vesicles is the bending rigidity of the membrane, which quantifies its resistance to changes in curvature ( Wubshet & Liu, 2023). It is well known that bending rigidity is influenced by a multitude of factors such as the hydrophobic tails’ chain length and saturation ( Rawicz *et al.*, 2000), the temperature ( Pan *et al.*, 2008b), the presence of charged lipids ( Dimova, 2014; Faizi *et al.*, 2019) the concentration of sugars or salts ( Dimova, 2014), and the presence of sterols like cholesterol ( Chen & Rand, 1997; Dimova, 2014; Gracià *et al.*, 2010a; Karal *et al.*, 2022).

Cholesterol is a crucial element of biological systems, constituting up to 50 mol% of the total lipid content of the membrane ( Karal *et al.*, 2020a), although its proportion varies between cell types. For example, lymphocyte membranes and certain metastatic cancer cells tend to exhibit lower cholesterol content. High cholesterol content generally increases membrane rigidity and decreases deformability, whereas membranes with lower levels are more flexible, enhancing their ability to deform and pass through the narrow intercellular spaces in tissues ( Szlasa *et al.*, 2020; Zhao *et al.,* 2019). For synthetic membranes, such as GUVs, the effect of cholesterol has been extensively debated in the literature. It is widely accepted that cholesterol has different effects on membranes depending on their constituting phospholipids ( Pan *et al.*, 2008a). The bending rigidity of saturated lipids membranes increases with cholesterol content, while in membranes composed of double unsaturated lipids, like dioleoylphosphatidylcholine (DOPC), the rigidity seems to be independent of cholesterol fraction ( Dimova, 2014; Gracià *et al.*, 2010a; Pan *et al.*, 2008a; Sorre *et al.*, 2009). Nonetheless, some evidence suggests the bending rigidity of DOPC membranes increasing alongside the cholesterol content as for saturated lipids membranes ( Ashkar *et al.*, 2019; Chakraborty *et al.*, 2020). These discrepancies likely reflect differences in measurement techniques: equilibrium approaches (e.g., flicker spectroscopy) capture the static bending modulus, while non-equilibrium methods (e.g., neutron spin echo, NSE) are more sensitive to membrane viscosity on nanosecond–microsecond timescales. Thus, some of the reported rigidity changes in DOPC–cholesterol membranes may in fact originate from altered membrane viscosity rather than changes in long-timescale elastic stiffness ( Heinrich & Nagle, 2025).

To assess the role of cholesterol in lipid membrane models, the effects were previously tested on LUVs (large unilamellar vesicles fabricated with the swelling method ( Chakraborty *et al.*, 2020; Gracià *et al.*, 2010b), revealing its influence on both the average size of vesicles and the membrane’s bending modulus ( Karal *et al.*, 2022). While this technique offers a simple and reliable method to obtain GUVs, its use is preferably avoided when aiming at encapsulating solutions and cargos within the vesicles. For the fabrication of artificial cells or microrobots, a more advantageous alternative is provided by the droplet transfer method ( Pautot *et al.*, 2003), which allows for higher efficiency of encapsulation of valuable internal GUV solutions, such as cell-free protein synthesis reactions ( Garenne *et al.*, 2021; Shimane & Kuruma, 2022) or microparticles ( Vutukuri *et al.*, 2020). Because the method is based on the dissolution of phospholipids into an oil phase and the formation of bilayers from their spontaneous adsorption at oil-water interfaces, it is important to consider that other organic molecules present in or added to the oil phase, like cholesterol, may or may not contribute to the membrane. Indeed, membranes of GUVs obtained via the droplet transfer method may have a cholesterol content that does not match that in the lipid solution. The actual cholesterol concentration can be significantly lower than the nominal one ( Weakly *et al.*, 2024). Consequently, cholesterol cannot be assumed to affect droplet transfer-made GUVs in the same manner it affects GUVs produced by other methods.

In this work, we study how the addition of cholesterol affects GUVs prepared with the droplet transfer method in terms of size distribution, temporal stability and deformability of the vesicles. We use the commonly adopted double unsaturated DOPC as the membrane’s main constituent and mineral oil as an organic solvent. Cholesterol is added in different proportions to the DOPC-oil lipid solution and vesicles are produced keeping all other process parameters unvaried. To assess the equilibrium deformation of the vesicles, we used a custom setup equipped with two permanent magnets, generating a uniform magnetic field, and an inner solution containing ferrofluid. Given the well-known stabilizing effects of cholesterol ( Zhang *et al.*, 2019), we also examined the impact of this lipid on the temporal stability of our membranes.

## Methods

The following materials were obtained from Sigma-Aldrich ^®^ (St. Louis, MO): DOPC (1,2-dioleoyl-sn-glycero-3-phosphocholine, in chloroform, C44H84NO8P, cat. 850375C-1G, CAS: 4235-95-4), sucrose ≥99.5% (GC) (α-D-Glucopyranosyl β-D-fructofuranoside, C12H22O11, cat. S9378-500G, CAS: 57-50-1), D-(+)-Glucose ≥99.5% (GC) (C6H12O6, cat. G8270-100G, CAS: 50-99-7), and cholesterol powder (3β-Hydroxy-5-cholestene, C27H46O, cat. C3045-5G, CAS: 57-88-5). The aqueous ferrofluid, containing approximately 20% by weight of 10 nm magnetite nanoparticles stabilized with citrate, was purchased from Qfluidics (France). Additionally, the neodymium N42 nickel-plated permanent magnets were sourced from Supermagnete (cat. Q-15-15-08-N). The experimental setup utilized for deformability tests was designed using Computer-Aided Design (CAD) software (e.g. Autodesk Fusion 360) and subsequently 3D printed in VisiJet M3 Crystal material (3D Systems Projet MJP 3600 HD Max). Gene Frames (250 µm thickness and 1 cm ^2^ area) for microscope glass slides were obtained from Thermo Fisher Scientific (cat. AB0576).

### Preparation of stock solutions

Two lipid stock solutions were prepared in mineral oil, one at 2.5 mg/mL (3.18 mM) DOPC and one at 0.79 mg/mL (2 mM) cholesterol. Lipids dissolved in chloroform were transferred onto a glass vial, then the chloroform was evaporated through a nitrogen flow, allowing the formation of a thin film of dried phospholipids. Finally, the mineral oil was added, and the lipids were dissolved by heating at 80°C for one hour. The stock was then stored at 4°C.

Two inner solutions and one outer solution were prepared using milliQ water. One inner solution was prepared with 240 mM sucrose, and the other with 1:2 (v/v) ferrofluid:sucrose 240 mM, while the outer solution was prepared with 240 mM glucose. The solutions without ferrofluid were filtered with a 0.22 µm microfilter and then stored at +4°C.

### Preparation of GUVs through droplet transfer

GUVs were prepared following a modified version of the droplet transfer method ( Pautot *et al.*, 2003) described in our previous work ( Cecchi *et al.,* 2025). 200 µL of lipid stock solution were transferred in a 2 mL tube, sonicated for 10 minutes in a bath sonicator (Ultrasonic cleaner Brown Sonic 2510E-MTH), and cooled on ice for 15 minutes. An emulsion was then prepared by adding 10 µL of inner solution to the tube, vortexing the mixture for 25 seconds at 3000 rpm (Vortex WIZARD IR, VWR cat. # 444-0746) and chilling it for 10 minutes on ice. 300 µL of outer solution were transferred in a 1.5 mL tube and placed on ice. Then 100 µL of lipid stock solution were layered on top of the outer solution to allow for the spontaneous formation of a lipid monolayer at the water/oil interface. The emulsion was then layered on top of the interface and centrifuged for 20 minutes at 3300g at 4°C. Finally, the oil phase was removed with a pipette and the liposomes were resuspended from the pellet.

For the preparation of GUVs with cholesterol, oil mixes composed of DOPC and cholesterol at different molar ratios were prepared following the previously reported analysis on GUVs made with the swelling method ( Karal *et al.*, 2022): 85:15, 71:29, and 60:40. DOPC concentration was kept constant at 1.25 mg/mL (1.59 mM) and the cholesterol concentration varied according to the molar ratio.

### Observation and data analysis of GUVs

After removing the oil phase with a micropipette and resuspending the liposomes, a 25 µL aliquot of the sample was placed onto a glass slide within a Gene Frame and covered with a coverslip. Samples were then observed in phase contrast through the inverted microscope Nikon Eclipse TE2000-U, equipped with a 20x/0.40 Ph1 ADL objective at a working distance (WD) of 3.1. Images of the entire frame area were captured. To investigate the effects of cholesterol on the size distribution of GUVs and to ensure consistent observation and statistical analysis of how cholesterol influences vesicle size distribution, triplicate samples of the four compositions (DOPC:Chol 100:0, 85:15, 71:29, and 60:40) were produced and analysed. To ensure comparability during analysis, approximately 120 images were acquired for each sample. For the analysis of vesicle stability, triplicate samples of DOPC:Chol 100:0, 85:15, 71:29, and 60:40 were stored overnight at 4°C and observed again on the following day. In this case, 100 images were captured for each sample. The acquired images were analysed with Fiji ImageJ ( https://imagej.net/software/fiji/). For each image, GUVs were identified, and their areas were measured using the tools provided by ImageJ. The collected data were analysed with a custom script implemented in the Julia language ( https://julialang.org/) that calculates the diameter of the GUVs from their area (assuming them as round), evaluates whether the differences between samples are statistically significant, fits the data to a distribution function, and plots the data and analysis results ( Roberti *et al.*, 2025). Finally, we have calculated the total surface area of all observed vesicles as the sum of the individual surface areas (assuming them as spherical), and compared the results obtained for as-prepared samples with those for overnight-stored samples. This comprehensive approach aims to assess the impact of cholesterol on the vesicle size distribution and stability.

### Observation and data analysis of magneto-GUVs

The characterization of magnetic GUVs and the observation of their deformability under the influence of an external magnetic field were carried out using a digital 3D optical microscope (Hirox RH-2000). To observe deformation, the sample was subjected to an external magnetic field generated by a pair of permanent magnets kept at specific distances by a custom 3D printed setup ( Roberti *et al.*, 2024). The magnets are oriented with the poles in the same direction and placed at equal distances to the observation area at the centre of the device. The superposition of the magnetic fields results in a homogeneous magnetic field in the observation area. 25 µL of the sample were placed on a microscope glass slide inside a Gene Frame and covered with a coverslip. All samples were initially observed without the application of a magnetic field. Subsequently, the glass slide was placed at the centre of the magnetis’ setup and the magnetic field intensity was varied by changing the distance between the magnets. Three different distances between the magnets were considered for our analysis: 13, 10 and 4 cm, resulting in magnetic fields of 2.0, 4.7, and 48.0 mT, respectively, which we named H1, H2 and H3 ( Roberti *et al.*, 2025). The images were subsequently analysed using ImageJ and each GUV was treated as an ellipse, collecting data on area, major axis, and minor axis. Data were then analysed with a script implemented in Julia ( Roberti *et al.*, 2025).

**Figure 1.**
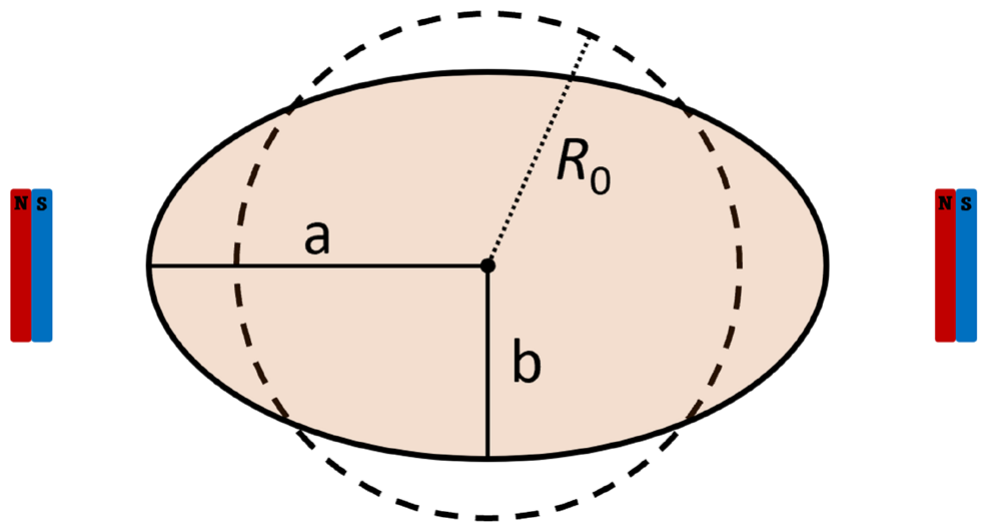
Schematics of the GUVs’ dimensions and the related rest radius.

The vesicles are assumed to have a spherical shape at rest and to achieve an ellipsoidal shape (namely a prolate spheroidal shape) under the action of a magnetic field. The long axis of the ellipsoid is aligned with the orientation of the magnetic field (see Figure 1).

Given the major and minor axis *a* and *b* of the spheroid, its volume can be calculated as $V=\frac{4}{3}\pi \;ab^{2}$ . Assuming that the volume of the vesicle does not change with the magnetic elongation, we can estimate the rest radius as $R_{0}=\sqrt[{3}]{ab^{2}}$ (and thus the rest diameter). This means, however, that the apparent surface of the vesicles increases, as the membrane spreads out. We thus calculate the surface area of a sphere having a radius equal to the rest radius, $S_{s}=4\pi R_{0}^{2}$ , and assumed it to be the rest apparent surface area of the vesicle. We also calculated the surface area of the vesicles in the observed prolate spheroidal shape as to *S _ps_* = 2 *π* *b* ^2^ (1 + ^*a*^/ *_be_* arcsin *e*), where *e* ^2^ = 1 – ^*b* ^2^^/ _*a* ^2^_. As we are interested in the effect of the lipidic composition of the membrane on vesicles deformability at equilibrium, we then evaluated the surface area deformation as *σ* = *S*_ps_/ *S*_s_ – 1. Statistical significance tests on *σ* are performed assuming a lognormal distribution.

### Statistical analysis

Statistical analyses were performed to evaluate the influence of cholesterol content on the size distribution and temporal stability of vesicles as well as on their magnetically induced deformation. Fitting the diameter data to lognormal distributions, we also calculate mode (i.e. the peak of the distribution), median, and percentiles of the distribution of each sample. Assuming lognormal distributions of the values of diameters and of σ, the statistical significance tests (F-test and t-test) are not performed on the actual data but on their natural logarithm (which have normal distributions). Size distribution analyses were carried out across samples with different cholesterol concentrations at both t _0_ and after overnight incubation using two-sample t-tests (see GUVs_concentration_stability.csv, Underlying Data). Stability was further assessed, reporting the peak, median and number of GUVs at t _0_ and after overnight incubation for each concentration. Two-sample t-tests were performed between samples at pristine and overnight stored samples (corresponding p-values are reported in GUVs_stability_T0vsON.csv, Underlying Data). Moreover, the minimum detectable ratio between groups (σ _1_/σ _2_), as determined by the log-transformed *t*-test at 80% power, was incorporated into the analysis to define the smallest effect size that could be reliably identified under our experimental conditions (see GUVs_deformability_stat, Underlying Data).

## Results

### Size distribution

**Figure 2.**
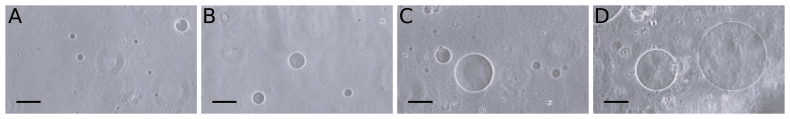
Representative phase-contrast images of **A**) DOPC:chol 100:0, **B**) DOPC:chol 85:15, **C**) DOPC:chol 79:21, **D**) DOPC:chol 60.40. Scale bars = 50 μm.

**Figure 3.**
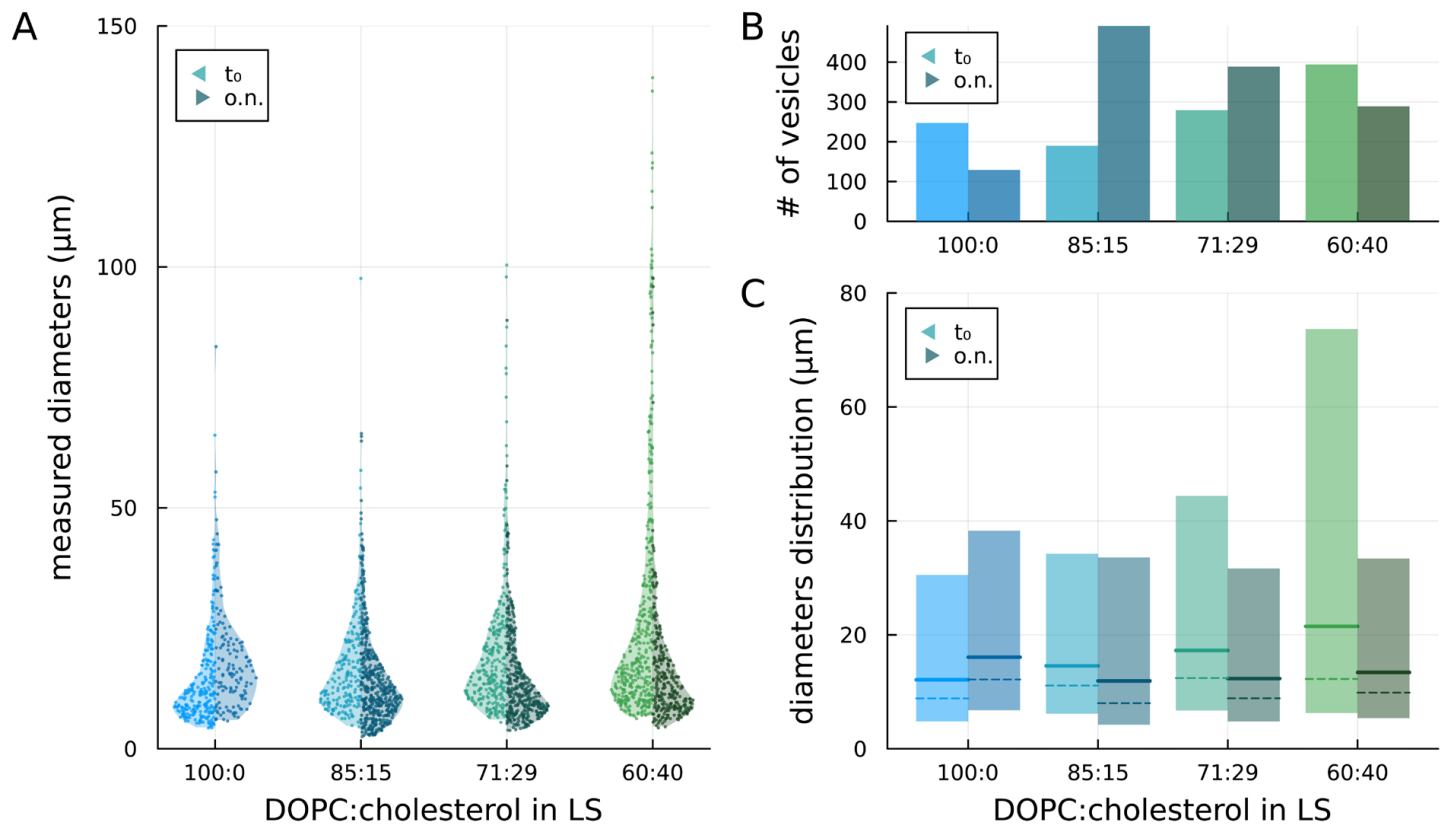
Comparison of vesicles prepared with lipid solutions (LSs) at different concentrations of cholesterol, as prepared (t _0_) and after overnight storage (o.n.). **A**) measured diameters of the vesicles; **B**) number of observed vesicles; **C**) median (solid lines), mode (peak – dashed lines) and 5 ^th^ to 95 ^th^ percentile (coloured areas) of the lognormal distributions fitted to the diameters data.

We prepared GUVs by the droplet transfer method at different DOPC:cholesterol ratios, namely 100:0 (no cholesterol), 85:15, 71:29, and 60:40, in the lipid solution ( Figure 2). Right after preparation (t _0_), we observed 247, 190, 279, and 394 vesicles, respectively, in the sample aliquot (see Figure 3B left side / bright colour). Assuming a lognormal distribution of the diameters within each sample (see Methods), we found that the dimensional differences between all combinations of samples are statistically significant ( *p* < 0.005; *p* values are reported in Underlying Data). Moreover, as the concentration of cholesterol in the lipid solution increased, the maximum observed diameter of vesicles increased.

Fitting lognormal distributions to the diameters data, we found that the median diameter (geometric mean of the distribution) increased with the content of cholesterol in the lipid solution consistent with what was described in previous studies ( Karal *et al.*, 2020b; Karal *et al.*, 2022). The estimated median diameters are 12.1, 14.5, 17.3 and 21.5 µm, respectively for 100:0, 85:15, 71:29, and 60:40 distributions (see Figure 3C, solid lines – left side / bright colour). The coloured bars in Figure 3C represent the dimensions of vesicles within the 5 ^th^ and 95 ^th^ percentile of the distributions: it can be noticed that the main difference among samples with increasing concentrations of cholesterol in the lipid solution is the increase in the 95 ^th^ percentile value and, thus, in the width of the distribution.

### Temporal stability

To investigate the impact of cholesterol on the stability of the GUV membrane, samples were stored overnight at 4°C and observed at the phase-contrast microscope the following day. For each sample, we recorded the number of vesicles observed in a new aliquot and their dimensional distribution (see Figure 3 – right sides / dark colours). Among the overnight-stored aliquots, the sample 100:0 has a significantly different size distribution from all cholesterol-containing samples ( *p* < 0.005), whereas all cholesterol-containing samples present no statistically significant differences among them ( *p* values are reported in Underlying Data). When cholesterol was absent (sample 100:0), the total number of observed GUVs almost halved and the median diameter from the fitted lognormal distribution increased with a general shift towards larger diameters. Conversely, for the cholesterol-containing samples, a general shift towards smaller diameters was observed with a decrease in the upper limit of the distribution (95 ^th^ percentile) compared to the no-cholesterol samples. With the highest cholesterol concentration (60:40), both the number of vesicles and the median diameter decreased overnight. For the intermediate cases (85:15 and 79:21), instead, the number of GUVs increased while the median diameter decreased. In particular, for the 85:15 samples, the number of vesicles more than doubled and the median diameter decreased. The differences in size distributions between the pristine and overnight aliquot are statistically significant for all samples ( *p*<0.005; *p* values are reported in Underlying Data).

Finally, we estimated the total surface area *A _s_* of vesicles and observed a decrease in all the samples (especially in those with the highest cholesterol concentration) except for the 85:15 sample where, surprisingly, the total surface area almost doubled overnight ( Table 1).

**Table 1.** Overnight variations in GUV populations. Median diameters were calculated assuming a lognormal distribution (see Methods). All values in the table represent cumulative results from triplicate experiments.

DOPC: cholesterol	Median diameter at t = 0	Median diameter after overnight storage	Count at t = 0	Count after overnight storage	$\frac{A_{s}^{o.n.}}{A_{s}^{t\;=0}}$
100:0	12.1 µm	16.1 µm	247	129	82 %
85:15	14.5 µm	11.9 µm	190	491	198 %
71:29	17.3 µm	12.3 µm	279	389	66 %
60:40	21.5 µm	13.4 µm	394	289	20 %

### Deformation under magnetic fields

**Figure 4.**
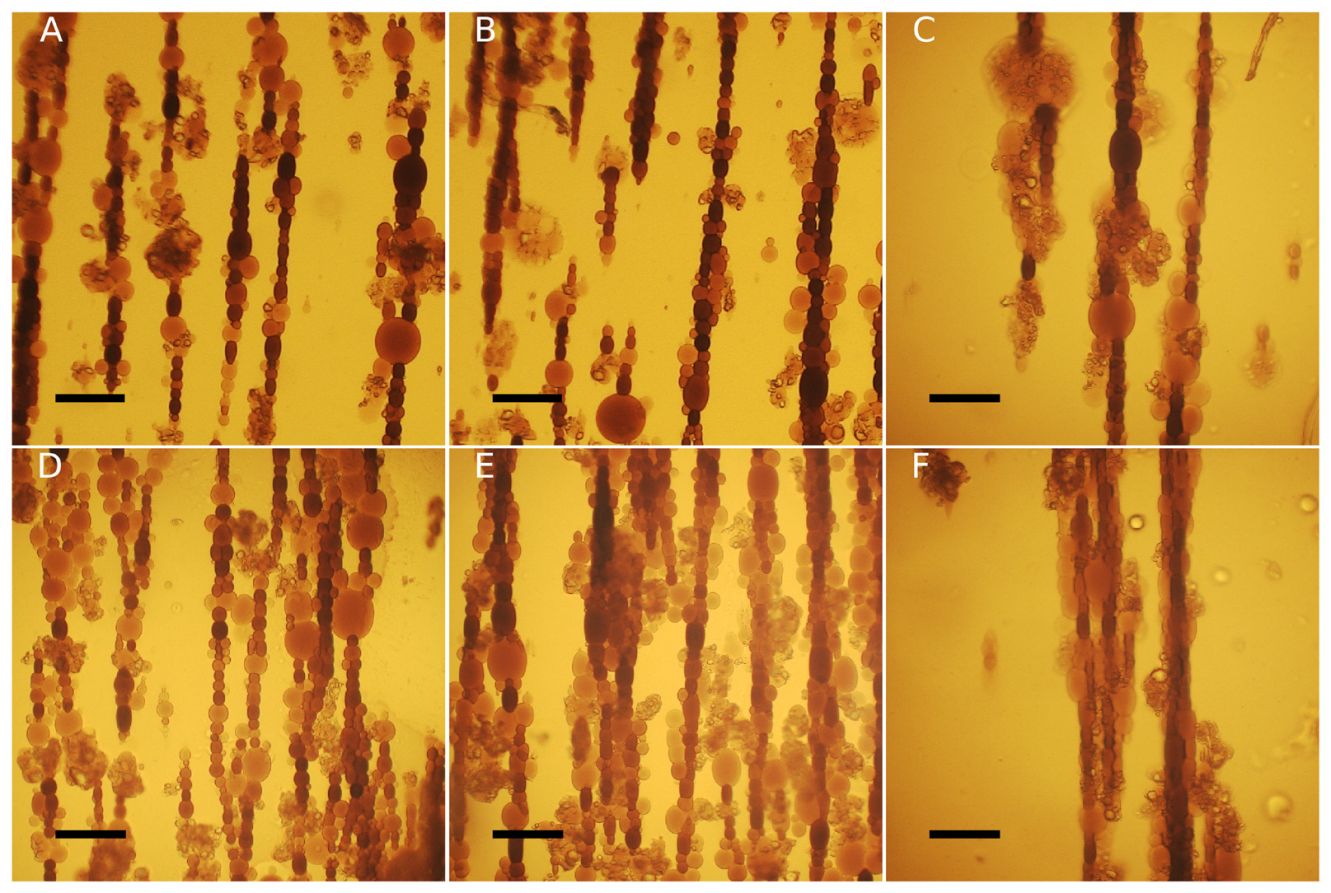
Bright-field optical microscope images depict DOPC:chol 60:40 ( **A**– **C**) and DOPC:chol 100:0 ( **D**– **F**) undergoing deformation at different magnetic field intensities: **A** and **D**: H1; **B** and **E**: H2; **C** and **F**: H3. Scale bars: 100 μm.

**Figure 5.**
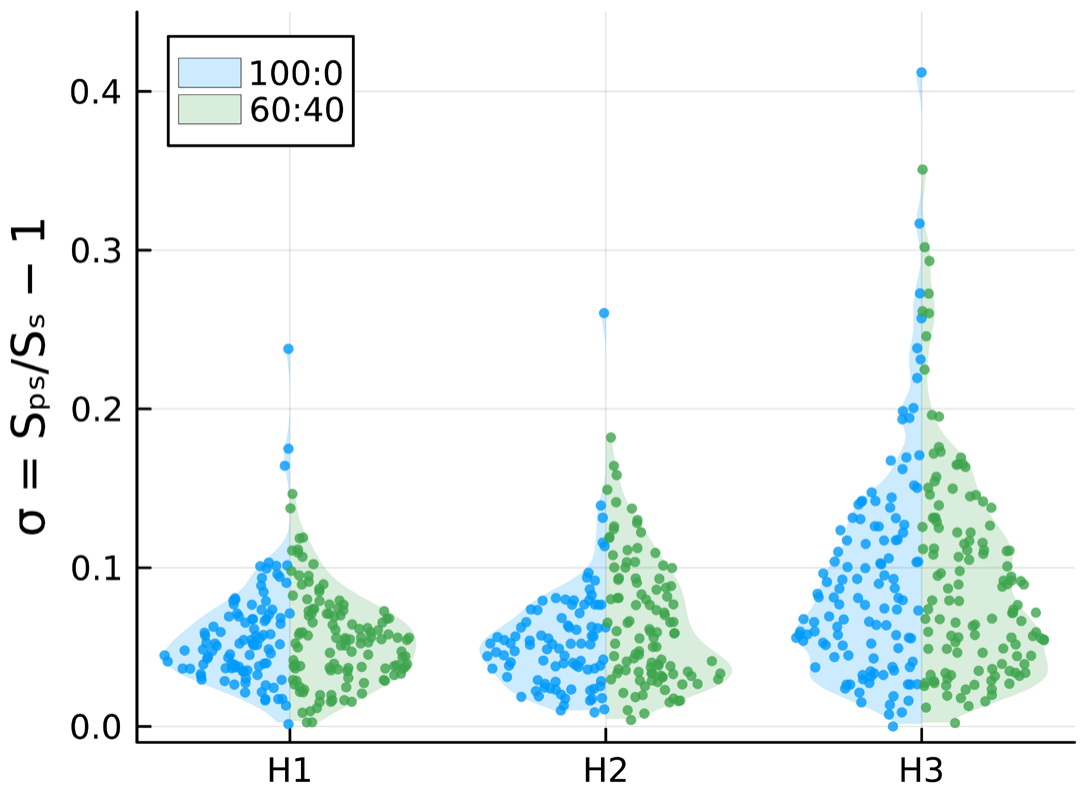
Comparison of *σ* values for DOPC:chol 100:0 and 60:40 under increasing magnetic fields.

As ferrofluid-loaded vesicles undergo magnetic field-dependent elongation ( Nuñez-Magos *et al.*, 2021), we exposed GUVs to uniform magnetic fields in order to investigate a potential correlation between their deformability and the presence of cholesterol in their membrane (see Observation and data analysis of magneto-GUVs). Following the application of the magnetic field, the vesicles exhibit a prolate shape, elongating in the direction of the field. Additionally, they tend to aggregate due to magnetic dipole-dipole interactions thus assembling in chains, as shown in Figure 4. For each measured vesicle, we calculated the surface area deformation *σ* with respect to an ideal spherical rest configuration. The results, reported in Figure 5, show that, as expected, the deformation increases with the intensity of the applied magnetic field, with some vesicles undergoing a surface area deformation of about 40% at the highest-intensity magnetic field. Although it can be observed in Figure 5 that 100:0 samples have one or few outliers with large deformations, whereas this is not observed in the 60:40 samples, the differences in surface area deformation between the two samples are not statistically significant ( *p*<0.005; p values are reported in Underlying Data). This suggests that cholesterol does not affect the mechanical properties of lipid bilayers. Nonetheless, given the observed variance in values of σ and aiming at a statistical power of 80%, we would have been able to detect ratios between median values of σ of about 1.4 or more (see Underlying Data).

## Discussions and conclusions

Cholesterol is a crucial component of cellular membranes that influences their physical properties by modifying the spatial organisation of phospholipid acyl chains. Due to this significance, cholesterol has been employed in the production of GUVs, both as models in biophysics studies and as semi-permeable shells for artificial cells. The effect of cholesterol on vesicles average size and their membrane’s bending modulus has been previously analysed for vesicles prepared through the swelling method ( Chakraborty *et al.*, 2020; Gracià *et al.*, 2010b; Karal *et al.*, 2022). In our study, we evaluated the effect of cholesterol on GUVs prepared with the droplet transfer method, where phospholipids and cholesterol are dissolved in an oil phase and the bilayer’s assembly is mediated by the water/oil interface. GUVs produced using the droplet transfer method may contain significantly less cholesterol than the lipid solutions from which they are prepared, especially when compared to other production techniques such as electroformation or gentle hydration. For this reason, we did not expect the membrane composition to match that of the original lipid solution as previously reported ( Weakly *et al.*, 2024). Nonetheless, we expect the cholesterol content in the membrane to depend on that in the lipid solution. Given the uncertain partitioning of cholesterol between the oil and the lipid bilayer, we aimed to estimate cholesterol's influence on droplet transfer-based GUVs, if any.

To this end, we examined the dimensional distribution, temporal stability, and deformability of GUVs with various lipid compositions produced through the droplet transfer method. Specifically, we fabricated vesicles with DOPC and different cholesterol ratios (100:0, 85:15, 71:29, 60:40). Our observations revealed that an increase in cholesterol ratio correlates with a higher average diameter of GUVs. This aligns with the previously observed role of cholesterol in lipid bilayers. Indeed, due to its hydrophobicity, cholesterol is likely embedded within the bilayer, reducing the *trans-gauche* isomerization of the neighbouring lipid acyl chains. This decreases their dynamics and fluidity, thereby stabilizing the membranes and leading to larger-diameter vesicles ( Yang *et al.*, 2016). We also analysed how cholesterol affects the temporal stability of GUV membranes. All four samples were stored overnight and observed the following day using a phase contrast microscope. GUVs without cholesterol (DOPC:Chol 100:0) were fewer in number but larger in size compared to the freshly prepared samples (t _0_), possibly indicating a tendency to fusion. On the other hand, cholesterol-containing GUVs undergo a reduction of the mean diameter overnight, more evident in the sample with the highest cholesterol concentration. The 85:15 and 71:29 samples showed an increase in vesicle numbers compared to t _0_, while the 60:40 sample faced a reduction in numbers after an overnight incubation. Although we could not verify this, it is possible that in lower cholesterol samples larger GUVs split into smaller ones, while GUVs with the highest cholesterol content burst into either lipid aggregates or small liposomes undetectable at the microscope.

To investigate whether cholesterol addition enhances or hinders GUV deformability, we applied a magnetic field to provide mechanical stress to the lipid membranes. With the use of two permanent magnets and by encapsulating aqueous ferrofluid, we fabricated magnetic GUVs and exposed them to three intensities of a uniform magnetic field. The GUVs, initially spherical, became ellipsoidal under the magnetic field, with compression increasing alongside the magnetic field intensity ( Figure 5). Interestingly, GUVs remained stable and did not break under the magnetic field. Since it was not possible to observe any significant difference in GUVs deformability, we concluded that cholesterol does not have a clear influence on membrane rigidity, at least not to an extent observable with our technique. This finding is consistent with a recent study ( Heinrich & Nagle, 2025), which highlighted that equilibrium-based methods, such as the one applied here, typically reveal no changes in the bending modulus (Kc) of DOPC membranes upon cholesterol incorporation. This is likely due to the fact that equilibrium-based approaches primarily capture long-timescale elastic properties, whereas other techniques are susceptible also to changes in membrane viscosity that manifest on nanosecond–microsecond timescales.

In this work, we evaluated how cholesterol affects the size, stability, and deformability of DOPC GUVs obtained with the droplet transfer method. Given the debated stiffening effect of cholesterol on the bilayer, we investigated its role in our specific case. We can conclude that cholesterol, when added to DOPC vesicles prepared via the droplet transfer method, significantly increases the vesicle diameter but does not seem to have a drastic effect on their deformability. Nonetheless, it should be noted that the method we used to evaluate deformability does not allow us to assess potential local effects that could impact stability if proteins or channels were added. Taken together, our findings indicate that, under equilibrium conditions, cholesterol does not significantly alter the deformability of DOPC GUVs, a conclusion consistent with previous reports employing similar equilibrium techniques.

## Data Availability

Zenodo: Dimensions, stability and deformability of DOPC-cholesterol Giant Unilamellar Vesicles formed by droplet transfer https://doi.org/10.5281/zenodo.14267071 ( Roberti *et al.*, 2025) This dataset contains the following underlying data: “data” folder containing the data files with GUVs dimensions (acquired in ImageJ) GUVs_stability_T0.csv (t0, pristine sample).csv and GUV_stability_ON.csv (o.n., sample stored overnight): area in pixel GUVs_with_cholesterol_6040_1/2/3.csv: dimensions of magnetic GUVs made from 60:40 DOPC:cholesterol LS under magnetic fields H1/H2/H3 GUVs_without_cholesterol_1/2/3.csv: dimensions of magnetic GUVs made from 100:0 DOPC:cholesterol LS under magnetic fields H1/H2/H3 “results” folder containing outputs from the data analysis Figure3.svg and Figure5.svg: figures 3 and 5 of the article GUV_stability_T0_proc.csv and GUV_stability_ON_proc.csv: processed data with diameter in μm and surface area in μm ^2^. GUVs_stability_T0vsON.csv: fitted distribution parameters and total surface area, pristine vs overnight GUVs_concentration_stability.csv: *p* values for differences between samples from LSs at different concentrations, pristine and overnight GUVs_deformability_60_40.csv and GUVs_deformability_100_0.csv: dimensions of magnetic GUVs made from 60:40 DOPC:cholesterol LS under magnetic fields with calculated geometrical parameters GUVs_concentration_stability.csv: p values for differences between samples with and without cholesterol in terms of size and deformability; minimum detectable effect size “utilities” folder containing the script to calculate the magnetic field generated by two permanent magnets “magnetic_fields.jl” “size_distribution_stability.jl” main script for reproducing the data analysis related to size distribution and stability of GUVs “deformability.jl” main script for reproducing the data analysis related to the deformability of GUVs “Manifest.toml” and “Project.toml” computational environment files “_init.jl” utility script for reproducing the computational environment Zenodo: Dimensions, stability and deformability of DOPC-cholesterol Giant Unilamellar Vesicles formed by droplet transfer https://doi.org/10.5281/zenodo.14268212 ( Roberti *et al.*, 2024) This dataset contains the following extended data: “deformation_size” folder containing scatter plots of σ with respect to GUVs rest radii sd_deform_scatter_H1 sd_deform_scatter_H2 sd_deform_scatter_H3 “magnetic_device_support” folder containing the .stl files for 3D-printing the magnets-support of the magnetic device magnetic_device_support_part1 magnetic_device_support_part2 “size_distribution_magnetic” folder containing size distribution histograms comparing 100:0 DOPC:cholesterol and 60:40 DOPC:cholesterol samples, under the application of magnetic fields sd_magnetic_size_dist_allfields sd_magnetic_size_dist_H1 sd_magnetic_size_dist_H2 sd_magnetic_size_dist_H3 “size_distribution_T0vsON” folder containing size distribution histograms comparing pristine samples (t _0_) and samples after overnight storage (ON), for different DOPC:cholesterol ratios sd_size_dist_60_40 sd_size_dist_71_29 sd_size_dist_85_15 sd_size_dist_100_0

## Software availability

The software consists of two custom scripts, written specifically for analysing the data of this work, together with files for reproducing the computational environment required to run the scripts. The software files are provided with the data files in the Underlying Data dataset of this paper.

Archived software available from: https://doi.org/10.5281/zenodo.14267071

License: CC-BY 4.0

General use functions originally developed for analysing this work’s data are collected in a GitHub repository.

Source code available from: https://github.com/microrobotlab/GUV-dimensional-analysis

Archived software available from: https://github.com/microrobotlab/GUV-dimensional-analysis/releases/tag/v1.0

License: OSI approved open license software is under MIT License

The scripts and repository content require the Julia language ( https://julialang.org/) to run.

Source code available from: https://github.com/JuliaLang/julia

Archived software available from: https://github.com/JuliaLang/julia/releases/tag/v1.10.8

License: OSI approved open license software is under MIT License
