# Supplementary material for: Dimensions, stability, and deformability of DOPC-cholesterol giant unilamellar vesicles formed by droplet transfer
Source: Open Res Eur. 2025 Oct 7;5:77. Originally published 2025 Mar 24. [Version 3] doi: 10.12688/openreseurope.19149.3 (PMC12326166; doi:10.12688/openreseurope.19149.3)
Supplement: Peer review history

# Reviewer report: Reviewer response for version 2

**DOI:** 10.21956/openreseurope.22736.r57440

**Published:** 2025-08-06

**Authors:** Nikoleta Ivanova

**Affiliations:** 1 University of Chemical Technology and Metallurgy, Sofia, Bulgaria

I have no comments on the draft manuscript.

Is the study design appropriate and does the work have academic merit?

Partly

Is the work clearly and accurately presented and does it cite the current literature?

Yes

If applicable, is the statistical analysis and its interpretation appropriate?

Yes

Are all the source data underlying the results available to ensure full reproducibility?

Yes

Are the conclusions drawn adequately supported by the results?

Yes

Are sufficient details of methods and analysis provided to allow replication by others?

Yes

Reviewer Expertise:

Molecular Dynamics Simulation, Physical Chemistry, Computational Chemistry

I confirm that I have read this submission and believe that I have an appropriate level of expertise to confirm that it is of an acceptable scientific standard.

# Reviewer report: Reviewer response for version 2

**DOI:** 10.21956/openreseurope.22736.r57438

**Published:** 2025-08-05

**Authors:** Martín E. Villanueva

**Affiliations:** 1 Université libre de Bruxelles, Bruxelles, Belgium

The authors have appropriately cited relevant references throughout the manuscript. Additionally, they have improved the statistical analysis, confirming the validity of their results and supporting the conclusions. I have no further comments on the current version of the paper.

Is the study design appropriate and does the work have academic merit?

Yes

Is the work clearly and accurately presented and does it cite the current literature?

Yes

If applicable, is the statistical analysis and its interpretation appropriate?

Partly

Are all the source data underlying the results available to ensure full reproducibility?

Yes

Are the conclusions drawn adequately supported by the results?

Partly

Are sufficient details of methods and analysis provided to allow replication by others?

Yes

Reviewer Expertise:

Membrane biophysics, Biomembrane models, Physical Chemistry, Nanotechnology.

I confirm that I have read this submission and believe that I have an appropriate level of expertise to confirm that it is of an acceptable scientific standard.

# Reviewer report: Reviewer response for version 1

**DOI:** 10.21956/openreseurope.20721.r56204

**Published:** 2025-07-23

**Authors:** David W. Everett

**Affiliations:** 1 Massey University, Palmerston North, Manawatu-Wanganui, New Zealand

**Dimensions, stability, and deformability of DOPC-cholesterol giant unilamellar vesicles formed by droplet transfer**

**Submitted to Open Research Europe**

 This manuscript reports on the effect of cholesterol on the membrane of giant unilamellar vesicles, an important topic for understanding digestibility of lipid structures in addition to the biological applications mentioned by the authors. Vesicle size, stability and deformability were measured at different ratios of DOPC to cholesterol using the droplet transfer method for constructing the vesicles. There was some recent work published on this topic that the authors may want to consider Zheng, H., Jiménez-Flores, R. & Everett, D.W. (2025). The impact of sphingomyelin and cholesterol on ordered lipid domain formation in the bovine milk fat globule membrane using artificial giant unilamellar vesicles as a model. JDS Communications, 6, 485-489, which may have some relevance to this current study. The introduction is well-written with a brief overview of why deformability is important in areas such as artificial cells and microrobotics. The impact of cholesterol is not yet fully understood, and this manuscript is a helpful addition to the scientific literature, although the conclusions are rather speculative based on the evidence found.

**Specific comments**

 The methods are appropriate and comprehensively described.

 The number of particles measured is in the hundreds for each measurement. Add a space between "279,190" as this may be construed as a much larger number rather than two numbers. Other methods, such as dynamic light scattering, measure very large numbers of particles and thus may be more representative of the population.

**Results**

 Were the increases in diameter (at time zero) at an increasing ratio of DOPC to cholesterol (Fig. 3C) statistically significant? There is no indication of this is the figure or in Table 1, although it is reported in the text as an overall p-value, not individual pairwise comparisons.

 For the 60:40 ratio, both vesicle numbers and size decrease overnight. Why is this? There appears to be missing membrane material that is not accounted for. The total GUV surface area should not change overnight if the surface structure remains the same. What accounts for the change in surface area? Could this be a result of measuring a relatively low number of particles that are not representative of the whole sample?

 Table 1: include a footnote to explain the last column heading.

 Figure 4: Did the total surface area include membrane from vesicles that are touching other vesicles? This would seem to be important, and may alter the conclusion drawn at the end of the results section. Did the ellipsoidal vesicle size change, compared to the spherical data presented in Table 1?

**Discussion**

 "Fusion" (also known as coalescence) is mentioned, but this is not the same as aggregation where two touching particles have separate membranes bound together. Which was occurring in this study? If it was fusion, there must be some unadsorbed DOPC and cholesterol present in the system.

 "Interestingly, GUVs remained stable and did not break under the magnetic field". This would preclude fusion from occurring.

 The discussion should consider the following paper in terms of the effect of cholesterol on deformability. This was not cited. Costello, A.L. & Alam, T.M. (2010). Investigating the impact of cholesterol on magnetically aligned sphingomyelin/cholesterol multilamellar vesicles using static 31P NMR. Chemistry and Physics of Lipids, 163, 506-513.

Is the study design appropriate and does the work have academic merit?

Yes

Is the work clearly and accurately presented and does it cite the current literature?

Yes

If applicable, is the statistical analysis and its interpretation appropriate?

Partly

Are all the source data underlying the results available to ensure full reproducibility?

Yes

Are the conclusions drawn adequately supported by the results?

Partly

Are sufficient details of methods and analysis provided to allow replication by others?

Yes

Reviewer Expertise:

Lipid vesicle structures

I confirm that I have read this submission and believe that I have an appropriate level of expertise to confirm that it is of an acceptable scientific standard, however I have significant reservations, as outlined above.

# Response

**Published:** 2025-08-06

**Authors:** Elisa Roberti

**Affiliations:** BioRobotics Institute, Sant'Anna School of Advanced Studies, Pisa, Tuscany, Italy

1. We thank the reviewer for pointing out the error in the text; we have revised it accordingly (version 3). Regarding the use of DLS, our GUVs are too large to be measured with this technique. Therefore, suitable techniques are microscopy and flow cytometry.

 2. In version 2 of the manuscript, we revised the statical analysis and added a CSV file in the Underlying Data containing a pairwise comparison of the concentrations at t0 and to.n..

 3. It is likely that the observed decrease in both number and size overnight is due to the splitting of vesicles into nanoliposomes, which cannot be detected by microscopy and cannot therefore be analyzed.

 4. The total surface area is calculated based on liposomes without ferrofluid, which are not subjected to deformation induced by the magnetic field. These are isolated GUVs that do not come in contact with each other. Regarding the size of GUVs containing ferrofluid and thus subjected to the deformability test, the Extended Data contain graphs showing the equivalent diameters of the magnetic GUVs, calculated from fitted ellipses under the assumption of prolate spheroidal shape. These graphs indicate that, in general, GUVs containing ferrofluid tend to be larger than empty ones.

 5. We expect that fusion events between GUVs could occur over long timescales, particularly during extended storage or incubation, even though we did not observe fusion within the short timescale of the deformability experiments. Therefore, fusion does not influence deformability measurements, and the observed mechanical responses can be attributed to the intrinsic properties of the individual vesicles. Nonetheless, we believe that fusion could explain the change in number and size of vesicles during overnight storage.

 6. The suggested article specifically discusses multilamellar vesicles containing sphingomyelin, which is not present in our system. For this reason, we do not consider it relevant to our study.

 7. In version 3 of the manuscript we have included a footnote to explain the last column heading of Table 1.

# Reviewer report: Reviewer response for version 1

**DOI:** 10.21956/openreseurope.20721.r55563

**Published:** 2025-07-21

**Authors:** Josep Julve

**Affiliations:** 1 Institut de Recerca Sant Pau (IR SANT PAU), Barcelona, Spain

Technical issues:

 1- Methodology: no statistical analysis subsection has been considered in the methodology. why? How many independent replicates were done?

 2- How many independent replicates were done in each pack of experiments (grouped in figures/tables); for instance, in figures 2 and 3, the authors showed representative images and collected size data but did not mention how many independent experiments were done. The authors did not mentione how was the reproducibility in this case. 

 2-Why the assesment of physical characteristics of GUVs was not carried out under room or physiological temperatures o/n? May the temperature influence the indicated physical characteristics of GUVs? Possibly, this could be at least considered as one of the main limitations of this study. Please discuss

 3- May the authors consider the possibility to use alternative tools to detemine sucha as flow cytometry to assess number and size dstribution of newly-generated GUVs?

 4- Were the ratios between cholesterol and POPC moietis within the range of physiological? 

 5- A schematic flux diagram (new figure) could be suitable to visualize the technical protocol to obtain GUVs.

 6- Methodology, suggestion: trypan blue dye is commonly used in cell cultures to assess cell viability; however, this dye is also a suitable fluorochrome for staining and visualize GUs under confocal microscopy allows high resolution and contrast, and enables the creation of sharp, high-resolution images and 3D reconstructions, that would enrich the analysis of the geometry of generated GUVs.

 7- In light of previous studies, the authors used the double unsaturated DOPC as a representative phospholipid to study the impact of increasingly higher concentration cholesterol on membrane characteristics; it is not discussed the whether other phospholipids may behave similarly in the formation of GUVs and to study the influence on GUVs across cholesterol concentration.

Is the study design appropriate and does the work have academic merit?

Partly

Is the work clearly and accurately presented and does it cite the current literature?

Yes

If applicable, is the statistical analysis and its interpretation appropriate?

No

Are all the source data underlying the results available to ensure full reproducibility?

No

Are the conclusions drawn adequately supported by the results?

Yes

Are sufficient details of methods and analysis provided to allow replication by others?

Yes

Reviewer Expertise:

lipid metabolism

I confirm that I have read this submission and believe that I have an appropriate level of expertise to confirm that it is of an acceptable scientific standard, however I have significant reservations, as outlined above.

# Reviewer report: Reviewer response for version 1

**DOI:** 10.21956/openreseurope.20721.r56207

**Published:** 2025-07-21

**Authors:** Martín E. Villanueva

**Affiliations:** 1 Université libre de Bruxelles, Bruxelles, Belgium

In the present paper, the authors have investigated the influence of cholesterol on the properties of Giant Unilamellar Vesicles (GUVs) prepared via the droplet transfer method using varying DOPC:cholesterol ratios. They systematically examined how cholesterol content affects GUV size distribution, temporal stability, and deformability. The study combines phase-contrast microscopy, magnetic deformation assays using ferrofluid-loaded vesicles, and quantitative image analysis to assess these physical characteristics. The results show that increasing cholesterol content leads to larger median diameters and broader size distributions, while stability over time seems affected in a composition-dependent manner. The authors conclude that cholesterol does not significantly affect vesicle deformability under magnetic stress and propose that adjusting the lipid composition in the oil phase offers a simple yet effective strategy to tune GUV properties.

 While the proposed approach has certain potential, I have a few concerns on how the results are displayed and interpreted. I will go through them point by point:

1. The bar plot indicating number of vesicles at t _0_ and overnight in Figure 3 indicates that the lowest number of vesicles is presented at t _0_ by the 85:15 DOPC:Cho mixture. However, if one follows the corresponding part of the text in the Results section, it is understood that the 71:29 mixture displays the lowest amount of vesicles. One of them should be corrected.
2. Concluding that cholesterol has no clear influence on membrane rigidity is somewhat problematic, even if this appears to be the case for the specific system studied. As the authors are aware, cholesterol is well known to rigidify lipid bilayers, a phenomenon extensively documented in the literature. In fact, one of the cited references (Karal et al. 2022) clearly highlights how the bending modulus varies with different PC/cholesterol mixtures, both in bilayers and in GUVs, using various experimental techniques. Given this, the authors should discuss in greater detail the possible sources of the observed lack of difference in mechanical response. Could it be that the method used primarily probes area strain or surface deformation, which relates more closely to the area compressibility modulus (Ka)? In DOPC/cholesterol systems, cholesterol has a stronger effect on bending rigidity (κ) than on Ka. If the magnetic field primarily induces shape deformation rather than surface compression, this may explain the absence of a significant cholesterol effect. Additionally, if the GUV population varies in size, membrane pre-tension, or exact composition, such heterogeneity could obscure subtle mechanical differences due to cholesterol.
3. On the other hand, assuming that the method reliably supports the conclusion that incorporating up to 40 mol% cholesterol does not alter the mechanical properties of the GUVs, the authors provide little clarification on how the preparation protocol itself might influence these mechanical outcomes. This aspect is particularly relevant, as the method of GUV formation can significantly affect membrane structure and properties. Unlike electroformation, the droplet transfer method relies on monolayer-to-bilayer assembly, an interfacially driven process that may lead to different molecular arrangements. It is therefore plausible that this technique results in asymmetric membranes, even for relatively simple DOPC/cholesterol mixtures. As reported by Feigenson et al. (2022, BBA Acta), such asymmetry can promote the formation of induced ordered domains as a means to minimize midplane free energy. These structural differences could, in turn, impact measurements of bending rigidity and may help explain the absence of significant mechanical changes observed in the study.

Is the study design appropriate and does the work have academic merit?

Yes

Is the work clearly and accurately presented and does it cite the current literature?

Yes

If applicable, is the statistical analysis and its interpretation appropriate?

Partly

Are all the source data underlying the results available to ensure full reproducibility?

Yes

Are the conclusions drawn adequately supported by the results?

Partly

Are sufficient details of methods and analysis provided to allow replication by others?

Yes

Reviewer Expertise:

Membrane biophysics, Biomembrane models, Physical Chemistry, Nanotechnology.

I confirm that I have read this submission and believe that I have an appropriate level of expertise to confirm that it is of an acceptable scientific standard, however I have significant reservations, as outlined above.

# Response

**Published:** 2025-08-06

**Authors:** Elisa Roberti

**Affiliations:** BioRobotics Institute, Sant'Anna School of Advanced Studies, Pisa, Tuscany, Italy

1. We thank the reviewer for pointing out this error. The text has been updated in version 3.  2. We would like to clarify that, as also stated in the introduction, the literature does not present a unanimous view on the effect of cholesterol on membrane rigidity in synthetic systems. While many studies report a rigidifying effect of cholesterol, several others show no significant influence, or even contradictory results. These differences appear to depend not only on the method used to measure mechanical properties (e.g., micropipette aspiration, fluctuation analysis, electrodeformation) but also on the technique used to fabricate the vesicles (e.g., electroformation vs. gentle hydration). For this reason, we do not claim that cholesterol has no effect in general, but rather that in our specific system and under our experimental conditions, the effect, if present, is too small to be detected.  For what concern the relationship between surface deformation and Ka or κ, since not all membranes necessarily exhibit the same initial tension, σ can be correlated with both parameters, Ka and κ. 3. First, as clarified in version 2 of the manuscript, the actual cholesterol content in the membrane is likely lower than that in the initial lipid mixture. Therefore, we expect not to reach 40% cholesterol in the membrane of GUVs. Moreover, we did not explicitly account for the formation of asymmetric vesicles. Indeed, since the lipid solutions used for both the inner and outer leaflets were identical and composed of a single phospholipid (DOPC), we are reasonably confident that the final leaflet compositions are approximately symmetric.

# Reviewer report: Reviewer response for version 1

**DOI:** 10.21956/openreseurope.20721.r52406

**Published:** 2025-04-12

**Authors:** Nikoleta Ivanova

**Affiliations:** 1 University of Chemical Technology and Metallurgy, Sofia, Bulgaria

The main goal of the presented study was to research the stabilizing effect of cholesterol at different concentrations in bilayers composed of unsaturated lipid.

 The introduction includes information about lipid bilayers and their involvement in cell membranes. The functions of cholesterol and the effect of the presence of unsaturated lipids such as DOPC in lipid bilayers are discussed. The size and stability of Giant Unilamellar Vesicles (GUVs) were monitored with Phase-contrast microscopy, while the deformation was examined under magnetic field conditions.

 The main focus of my review is on the results and conclusions obtained in my scientific field of work related to MD simulations. The proposed work for review is well structured with clearly stated conclusions, but the studied effect is not fully confirmed by the applied method. MD simulations have reported the saturation (lack of changes) of some bilayer parameters at cholesterol content above 30%. It is noteworthy that the authors did not comment on the phase state of lipids at the relatively low temperature 4°C. This may also be the reason for the observed weak dependence of stability on cholesterol concentration.

 Suggested comments could be added to the Discussion and conclusions section.

Is the study design appropriate and does the work have academic merit?

Partly

Is the work clearly and accurately presented and does it cite the current literature?

Yes

If applicable, is the statistical analysis and its interpretation appropriate?

Yes

Are all the source data underlying the results available to ensure full reproducibility?

Yes

Are the conclusions drawn adequately supported by the results?

Yes

Are sufficient details of methods and analysis provided to allow replication by others?

Yes

Reviewer Expertise:

Molecular Dynamics Simulation, Physical Chemistry, Computational Chemistry

I confirm that I have read this submission and believe that I have an appropriate level of expertise to confirm that it is of an acceptable scientific standard, however I have significant reservations, as outlined above.

# Response

**Published:** 2025-07-17

**Authors:** Elisa Roberti

**Affiliations:** BioRobotics Institute, Sant'Anna School of Advanced Studies, Pisa, Tuscany, Italy

We thank the reviewer for the comment and for giving us the opportunity to clarify this concept. DOPC has a low transition temperature (Tm = -17°C). At 4°C, DOPC is above its Tm and thus remains in the liquid-disordered (Ld) phase. The transition temperature of phospholipids is influenced by the presence of cholesterol both in cell membranes and synthetic ones. At high temperatures, cholesterol tends to reduce membrane fluidity by interfering with the movement of phospholipids chains, making the membrane less permeable to small molecules. Instead, at low temperatures cholesterol prevents the membrane from becoming too rigid and stiff [1].   To date, no studies have been reported in the literature regarding the effect of cholesterol at 4 °C. Nevertheless, it can be hypothesized that cholesterol increases membrane fluidity by preventing the tight packing and crystallization of phospholipid fatty acid chains. Indeed, at low temperatures, the presence of cholesterol promotes greater lateral mobility of lipids, thereby inhibiting the formation of a rigid, ordered structure [2]. 

 In any case, this reasoning does not apply to our liposomes, which are composed of DOPC with a phase transition temperature (Tm) well below 4 °C. As a result, no phase transition occurs at this temperature, and cholesterol likely does not act to modulate the membrane from an ordered to a disordered state in this context. **Bibliography:**

 [1]    G. M. Cooper, “Structure of the Plasma Membrane,” in The Cell: A Molecular Approach. 2nd edition, Sinauer Associates, 2000. Accessed: Jun. 05, 2025. [Online]. Available: https://www.ncbi.nlm.nih.gov/books/NBK9898/

 [2]    E. L. CROCKETT, “Cholesterol Function in Plasma Membranes from Ectotherms: Membrane-Specific Roles in Adaptation to Temperature1,” American Zoologist, vol. 38, no. 2, pp. 291–304, Apr. 1998, doi: 10.1093/icb/38.2.291.
